# ASSISTED LIVING ADMINISTRATORS’ UNDERSTANDING OF REGULATORY REQUIREMENTS IN SEVEN STATES

**DOI:** 10.1093/geroni/igad104.0214

**Published:** 2023-12-21

**Authors:** Paula Carder, Kali Thomas, Lindsey Smith, David Reed, Philip Sloane, Sheryl Zimmerman

**Affiliations:** Portland State University Institute on Aging, Portland, Oregon, United States; Johns Hopkins University, Baltimore, Maryland, United States; Oregon Health & Science University, Portland, Oregon, United States; University of North Carolina at Chapel Hill, Chapel Hill, North Carolina, United States; University of North Carolina, Chapel Hill, North Carolina, United States; University of North Carolina at Chapel Hill, Chapel Hill, North Carolina, United States

## Abstract

Each state requires assisted living (AL) residences to employ an administrator whose job responsibilities include oversight of resident care, managing staff, and regulatory compliance. This study surveyed administrators of 151 AL residences in 7 states. These AL residences reflect 28 license types (e.g., based on level of care, dementia care services). Administrators were asked whether there were any regulations, policies or procedures that required their AL to do any of 11 health-related activities (e.g., staff able to check vital signs, advance directive on file, reporting change in condition to resident’s healthcare provider). Using health services regulatory analysis, we documented the applicable regulations for each of the 151 AL residences, then assessed whether administrator responses were congruent with the licensed requirements. Almost all (95%) administrators of AL residences governed by a licensure requirement regarding change in condition reported this policy in their residence. In contrast, less than half (48%) of administrators who work in an AL in our sample that state law requires to keep an advance directive on file for residents reported that they did not have a policy to do so. Over one-third (37%) of administrators who work in an AL required to have a policy of non-pharmaceutical treatment for residents with dementia said that they did not have this policy in place. Some administrators reported the presence of policies in the absence of state requirements, suggesting that these AL residences exceed minimum requirements. These results vary by AL license type both within and across states.

